# Nanoscale characterization of the temporary adhesive of the sea urchin *Paracentrotus lividus*

**DOI:** 10.3762/bjnano.9.212

**Published:** 2018-08-24

**Authors:** Ana S Viana, Romana Santos

**Affiliations:** 1 Centro de Química e Bioquímica e Centro de Química Estrutural, Departamento de Química e Bioquímica, Faculdade de Ciências da Universidade de Lisboa, Campo Grande 1749-016 Lisboa, Portugal; 2 Centro de Ciências do Mar e do Ambiente, Departamento de Biologia Animal, Faculdade de Ciências da Universidade de Lisboa, Campo Grande 1749-016 Lisboa, Portugal

**Keywords:** adhesive footprint, atomic force microscopy, nanomechanical properties, sea urchin, temporary adhesion

## Abstract

**Background:** Unlike the thin homogeneous films that are typical for adhesives produced by humans, biological adhesives present complex hierarchical micro- and nanostructures. Most studies on marine adhesives have focused on permanent adhesives, whereas the nanostructures of nonpermanent, temporary or reversible adhesives have only been examined in some organisms such as marine flatworms, barnacle cyprids, freshwater cnidaria and echinoderms such as sea cucumbers and sea stars. In this study, the first nanoscale characterization of sea urchin temporary adhesives was performed using atomic force microscopy (AFM).

**Results:** The adhesive topography was similar under dry and native (seawater) conditions, which was comprised of a honeycomb-like meshwork of aggregated globular nanostructures. In terms of adhesion forces, higher values were obtained in dry conditions, reaching up to 50 nN. Under native conditions, lower adhesive forces were obtained (up to 500 pN) but the adhesive seemed to behave like a functional amyloid, as evidenced by the recorded characteristic sawtooth force–extension curves and positive thioflavin-T labelling.

**Conclusion:** Our results confirm that like other temporary adhesives, the sea urchin adhesive footprint nanostructure consists of a meshwork of entangled globular nanostructures. Under native conditions, the adhesive footprints of the sea urchin behaved like a functional amyloid, suggesting that among its proteinaceous constituents there are most likely proteins with amyloid quaternary structures or rich in β-sheets. These results extend our knowledge on sea urchin adhesive composition and mechanical properties essential for the engineering of biomimetic adhesives.

## Introduction

Unlike the thin homogeneous films that are typical for adhesives produced by humans, biological adhesives present complex hierarchical micro- and nanostructures. Among marine adhesives, most studies have focused on permanent adhesives like those of mussels, barnacles and tubeworms, whereas the so-called nonpermanent, temporary or reversible adhesives have received much less attention. To our knowledge, the nanostructure of temporary adhesives has only been examined in a few organisms such as marine flatworms [1], barnacle cyprids [2–4], freshwater cnidaria [5] and echinoderms such as sea cucumbers [6] and sea stars [7–8]. This characterization was performed using atomic force microscopy (AFM), a technique that allows high-resolution images of soft biological materials to be obtained [9–10] as well as the nanomechanical properties. AFM is ideal for studying temporary marine adhesives (i.e., soft, hydrated materials, secreted in small quantities in a wet environment) under native conditions, requiring little or no sample preparation [8].

Parasitic marine flatworms such as *Entobdella solae* temporarily attach to fish skin using anterior pads located in the head [1]. Adhesion is brought about by interaction between two kinds of glandular secretions which are extruded together to form the adhesive [11]. The later consists of a network of highly insoluble proteinaceous fibres [12] that have been identified as amyloid fibres using the fluorochrome dye thioflavine-T, in addition to Raman spectroscopy and AFM [1]. AFM revealed a series of sawtooth mechanical responses reflecting the repetitive breaking of sacrificial bonds within an intermolecular β-sheet as protein is pulled from the surface of the adhesive fibrils and the “hidden length” of the amino acid chain is extended [1]. When probed directly in milli-Q water, pull-off forces around 63 ± 40 pN were needed to break these sacrificial bonds [1].

As for barnacle cyprid larvae they use glycoproteinaceous adhesive footprints to identify surfaces that meet the requirements for settlement. This is done by making use of two specialized antennules bearing a terminal adhesive disc that contains hypodermal glands filled with adhesive secretory granules [2]. The extruded adhesive was characterized by AFM in two species, *Semibalanus balanoides* [2–3] and *Balanus amphitrite* [4]. Cyprid adhesive footprints have a porous and fibrillar appearance, with nanofibrils of varying height (7–150 nm) and bundles of protein aggregates present in the network structure. The footprints probed in air (but kept moist with artificial seawater (ASW)) exhibited a mean pull-off force of 0.41 ± 0.20 nN [2]. Later on, adhesive footprints were probed in a moist environment with ASW using different AFM tips (Si_3_N_4_ and functionalized with CH_3_ terminal groups) and the obtained force–extension curves exhibited the characteristic sawtooth appearance for both tip types, but higher pull-off forces were obtained with CH_3_-tips (2.2 ± 0.4 nN) than with Si_3_N_4_-tips (1.1 ± 0.2 nN) [3]. As for the adhesive morphology of *B. amphitrite*, it was shown to be identical to *S. balanoides*, and force curves also presented the above-mentioned characteristic sawtooth appearance [4].

The freshwater cnidarian *Hydra magnipapillata* is a solitary polyp that lives temporarily attached during its whole lifecycle through its aboral adhesive disc, only detaching to search for better living conditions. The adhesive of *H. magnipapillata* is made up of proteins and neutral glycans, being produced, stored and delivered by a single cell type that encloses four types of secretory granules. When visualized with AFM, *H. magnipapillata* adhesive was found to be a homogenous meshwork with nanopores of about 0.5–1 μm, but no globular nanostructures were detected. The adhesion profile showed that pull-off forces were higher in the thinner areas of the footprint, reaching up to 66.4 nN [5].

Within echinoderms, sea cucumbers possess unique adhesive organs called Cuvierian tubules. The later are used for defence, entangling potential predators in a matter of seconds and allowing the sea cucumber to escape. During the elongation process, granular cells are exposed at the tubule surface and release their adhesive secretion composed of proteins and neutral sugars. However, the tissue integrity is compromised during the discharge and can only be used once. AFM was used to demonstrate that the adhesive from the tubules of the sea cucumber *Holothuria forskali* were made up of long fibres deposited on a homogeneous film. These fibres contain collagen as evidenced by their 70 nm periodicity, while the homogeneous film appears to be made up of globular nanostructures measuring about 70 nm in diameter. The adhesion map showed that pull-off forces were higher at the level of the homogeneous film (up to 17 nN) than at the level of the overlying fibres [6].

Common to all echinoderms is the presence of specialized adhesive hydraulic organs, called tube feet, which consist of a proximal extensible stem and a distal flattened disc [13]. The later releases an adhesive secretion that remains attached to the substrate as a footprint after detachment [14]. In sea stars, the adhesive is released through individual pores by two types of adhesive secretory cells found together in the disc epidermis [15]. The two secretions mix when extruded, producing adhesive footprints composed of proteins, neutral and amino sugars and uronic acid [16]. The adhesive nanostructure of sea stars was studied in two species, *Asterias rubens* [7] and *Marthasterias glacialis* [8]. AFM was employed to show that the adhesive footprints of *A. rubens* consist of globular nanostructures forming a meshwork deposited on top of a homogeneous film and their appearance was not different in dry or moist observation conditions [7]. The mesh height ranged from 70 to 200 nm and the diameter of the globular structures varied depending on the experimental conditions from 80–200 nm in moist-environment footprints and from 50–90 nm in dried ones, suggesting that drying may cause the adhesive material to shrink [7]. In *M. glacialis*, AFM showed that the adhesive material was multilayered, composed of a homogeneous film, a rough meshwork and a soft gel-like matrix with an embedded web of long interweaving fibres. However, a clear distinction between the homogeneous film and meshwork was not observed, nor were the globular nanostructures reported in *A. rubens*. A possible explanation might be the fact that the *M. glacialis* footprints were imaged under native conditions (ASW) while *A. rubens* footprints were imaged in either dry or moist (with distilled water) conditions. Accordingly, the height of the meshwork was higher in *M. glacialis* (200–500 nm) [8]. In terms of nanomechanical properties, no information is available for sea star adhesive footprints. In sea urchins, the adhesive is released not through pores like in sea stars, but at the apex of microvillar-like cell projections belonging either to one or two types of adhesive secretory cells (depending on the sea urchin species) [17]. In *Paracentrotus lividus* (which possess two cell types), the later occur together in the disc epidermis but not side-be-side like in sea stars, some being restricted to a small zone in the middle of the disc and others distributed in the rest of the central area of the disc epidermis [17]. The composition of the adhesive footprints has only been studied in *P. lividus* and was found to be composed of proteins and neutral sugars [18]. Although sea urchin tube feet and adhesive secretions have been extensively studied, its topography and mechanical properties at the nanoscale have never been investigated.

Therefore, the aim of the present work is to extend the current knowledge on sea urchin temporary adhesives by performing the first nanoscale characterization of the adhesive footprints of the sea urchin *Paracentrotus lividus*.

## Results and Discussion

**Figure 1:**
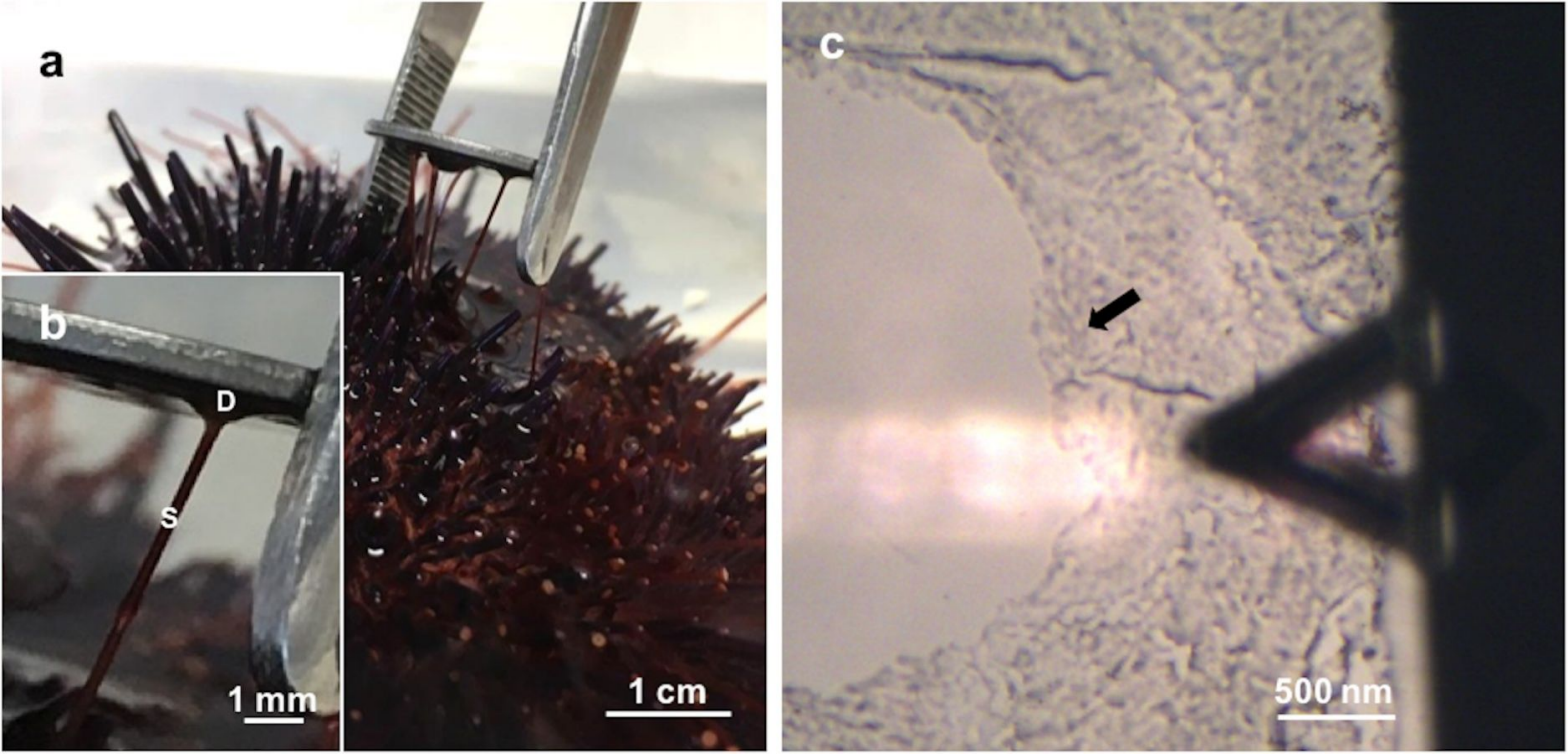
a) Collection of *Paracentrotus lividus* footprints on mica. b) Detailed view of a sea urchin tube foot attached to mica, showing the adhesive disc (D) and the stem (S). c) Optical microscopy (10×) illustrating the positioning of the moist adhesive footprint (indicated by the arrow) beneath the triangular-shaped AFM cantilever.

Consistent with previous descriptions, the adhesive footprints of the sea urchin *P. lividus* could be easily collected on mica (Figure 1a,b) and subsequently located using an optical microscope to be precisely positioned beneath the AFM cantilever (Figure 1c). The diameter of the adhesive footprints roughly corresponded to the size of the tube feet discs (±1 mm).

**Figure 2:**
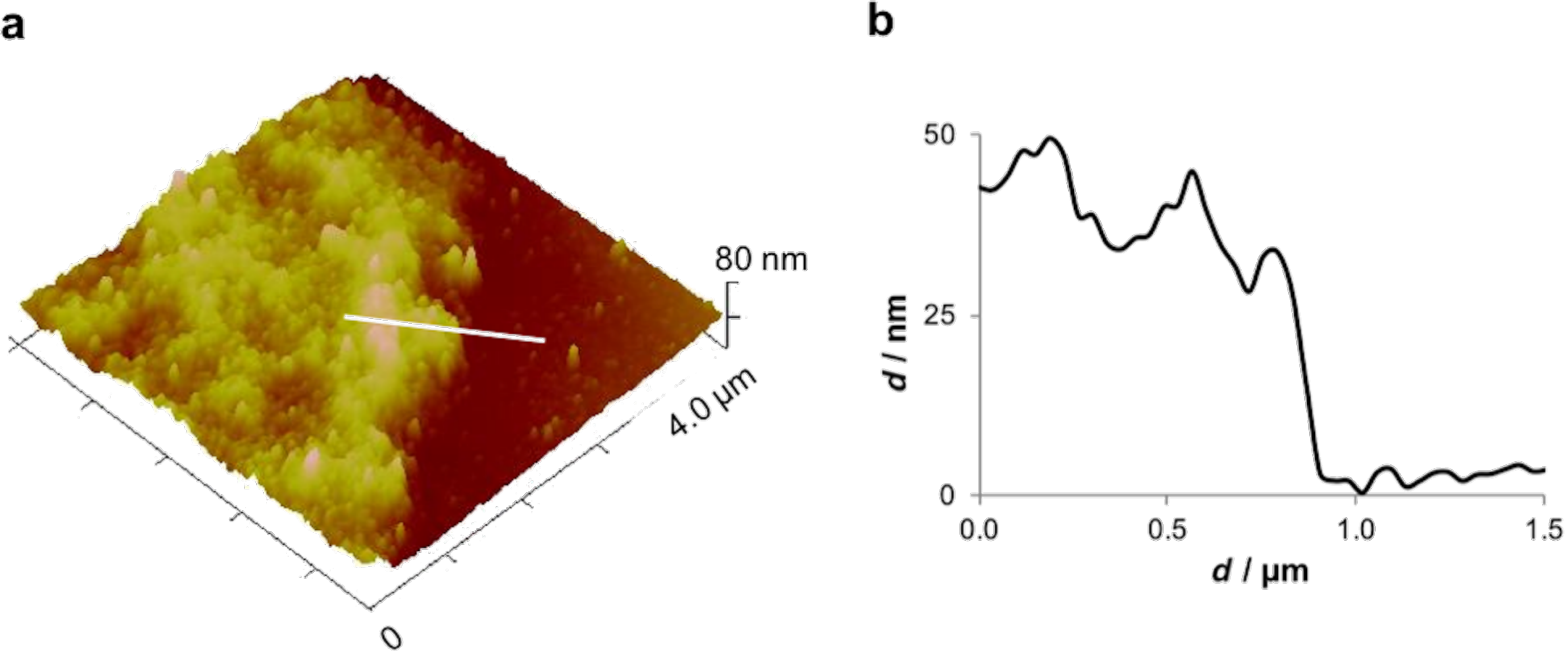
Peak force tapping AFM (PFT-AFM) image (a) and height profile (b) of *Paracentrotus lividus* moist footprints at the edge of the adhesive material. Image obtained with a ScanAsyst-Air probe.

The thickness of the adhesive footprint was quite variable, giving different aspects to the adhesive material. Therefore, thickness measurements by AFM were only performed in thin areas, at the edges of the footprint, where the adhesive material was not folded nor rippled. The obtained thickness values ranged between 30 and 50 nm (Figure 2). Previous results using interference-optical profilometry reported a thickness up to 100 nm for *P. lividus* moist adhesive footprints collected on glass [19]. These differences might be due to the fact that we are comparing measurements obtained with different techniques and on different substrates. In fact, in barnacles and sea stars, the thickness of the adhesive is clearly influenced by the surface on which it is deposited [2,7]. Previous studies with the barnacle *S. balanoides* showed that it is able to deposit footprints on different surfaces but with different thickness, R–NH_2_ terminated glass (positively charged surface) favouring the deposit of three times more adhesive than R–CH_3_ (hydrophobic surface) one [2]. In the sea star *A. rubens* the thickness of the adhesive is also influenced by the surface on which it is deposited, where more adhesive is deposited on glass and mica (two high-energy surfaces) than on teflon (a low-energy surface) [7].

**Figure 3:**
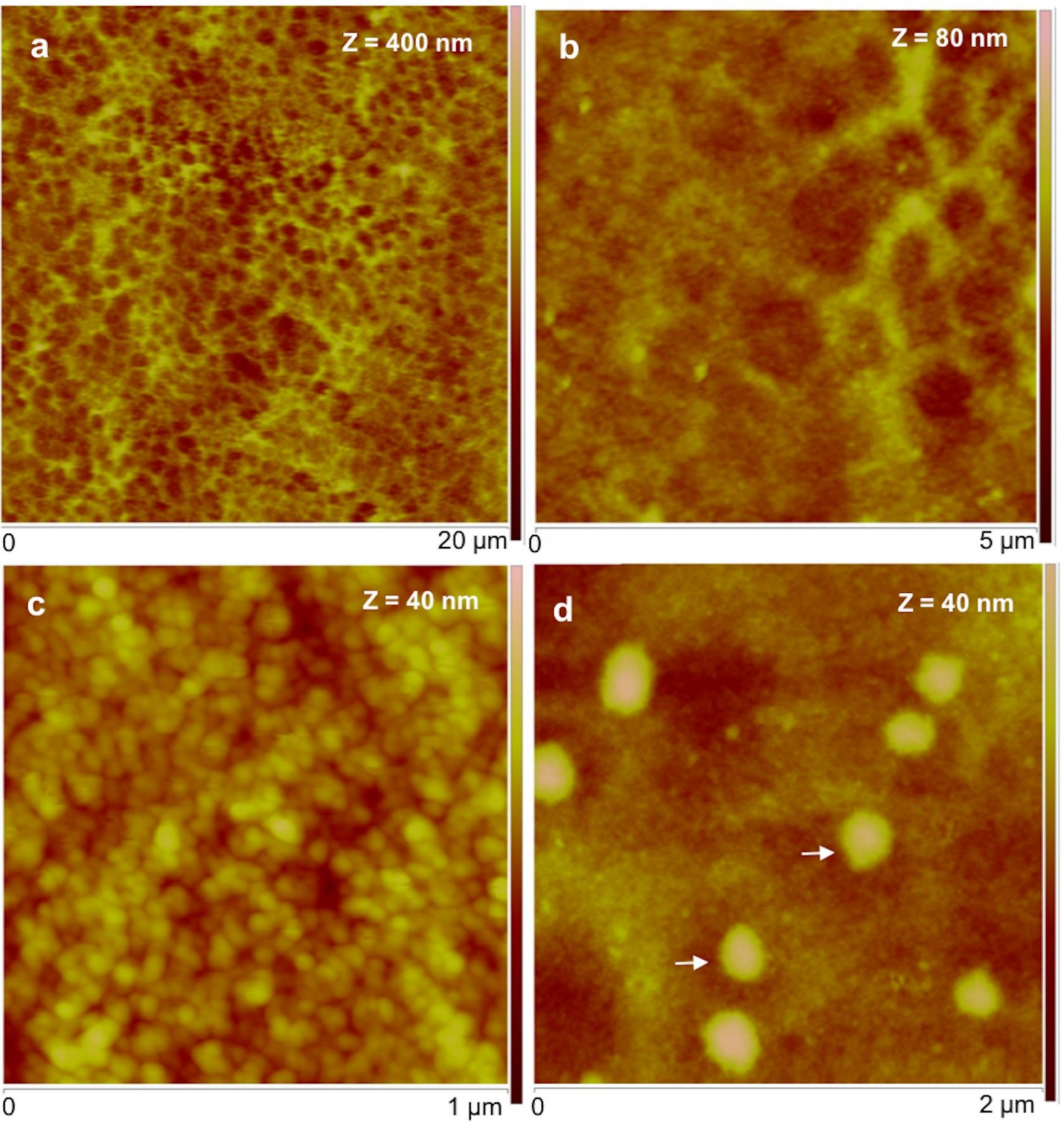
Peak force tapping AFM (PFT-AFM) images of moist adhesive material deposited by the tube feet of *Paracentrotus lividus* on mica. a) Height image observed in air. b) Higher resolution of the same area showing the honeycomb appearance of the meshwork. c) Detailed topography view showing that the meshwork is composed of aggregated globular nanostructures. d) Detailed topography view of a different area showing bigger globular structures denoted by white arrows. Images were obtained with a ScanAsyst-Air probe.

In order to understand the topography, *P. lividus* footprints were imaged using different probing methods (peak force tapping in air and fluid) and in various environments: dry, moist and under native conditions (ASW). In all the tested conditions, the adhesive material consisted of a meshwork formed by aggregated globular nanostructures. At low resolution, the meshwork has a honeycomb-like appearance (Figure 3a,b), but at higher resolution, it was possible to distinguish globular nanostructures with diameters ranging from 20 to 40 nm, most likely corresponding to aggregates of proteins (Figure 3c).

Common to all temporary adhesives is that the meshwork is made up of aggregates of globular nanostructures [1–8], which seem to be the elementary unit of bioadhesives. However, in sea urchin adhesives, these globular nanostructures were smaller (20–40 nm) than those observed in other temporary adhesives (70 nm for sea cucumbers [6], 50–200 nm for sea stars [7]).

The adhesive meshwork-like appearance is thought to be advantageous, providing economy of adhesive material, compliance, crack-stopping behaviour [20–21]; and the small size of the nanoparticles is thought to allow for increased contact between the adhesive and the surface, which is important to generate a large cumulative force for the adhesion process [22].

**Figure 4:**
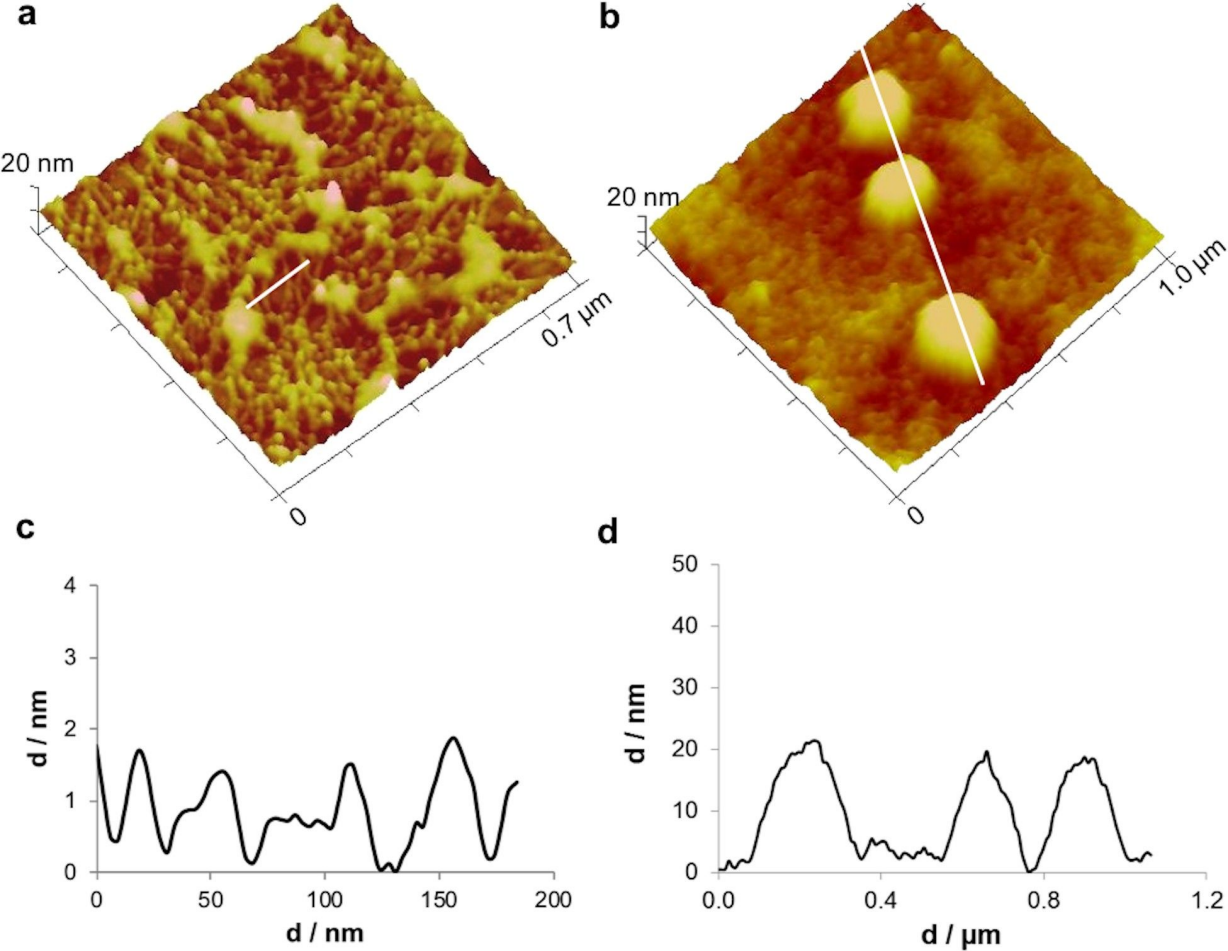
Peak force tapping AFM (PFT-AFM) 3D height images and profiles of small-sized areas of the moist adhesive material deposited by the tube feet of *Paracentrotus lividus* on mica. a) Height image and c) profile of the threads of aggregated globular nanostructures in a thinner peripheral area of the adhesive material. b) Height image and d) profile of the larger, globular structures in a thicker central area of the adhesive material. Images were obtained with a ScanAsyst-Air probe.

In thinner areas with less adhesive material, individual threads with a diameter of around 20 nm could clearly be distinguished (Figure 4a,c). These results are consistent with previous reports, describing the sea urchin footprint microstructure, observed by scanning electron microscopy (SEM), as a dense meshwork with smaller mesh-like areas (<1 μm) delimited by very fine fibrils (about 50 nm in diameter) [23]. Indeed, this dense meshwork of sea urchin footprints replicates the dense array of cell projections covering their tube foot disc surface [17] in the same way the loose meshwork observed in sea star footprints replicates the positioning of the secretory pores on their tube foot disc surface [7].

In the thicker, central areas of the adhesive, large granular structures with diameters ranging from 200 to 700 nm were often observed (Figure 3d and Figure 4b,d) on top of a uniform globular film. Given that *P. lividus* adhesive secretory granules were reported to have diameters ranging from 300–700 nm [17], it is clear from the shape, size, location and abundance that these bigger globular nanostructures correspond to adhesive secretory granules that remained attached to the adhesive secretion.

**Figure 5:**
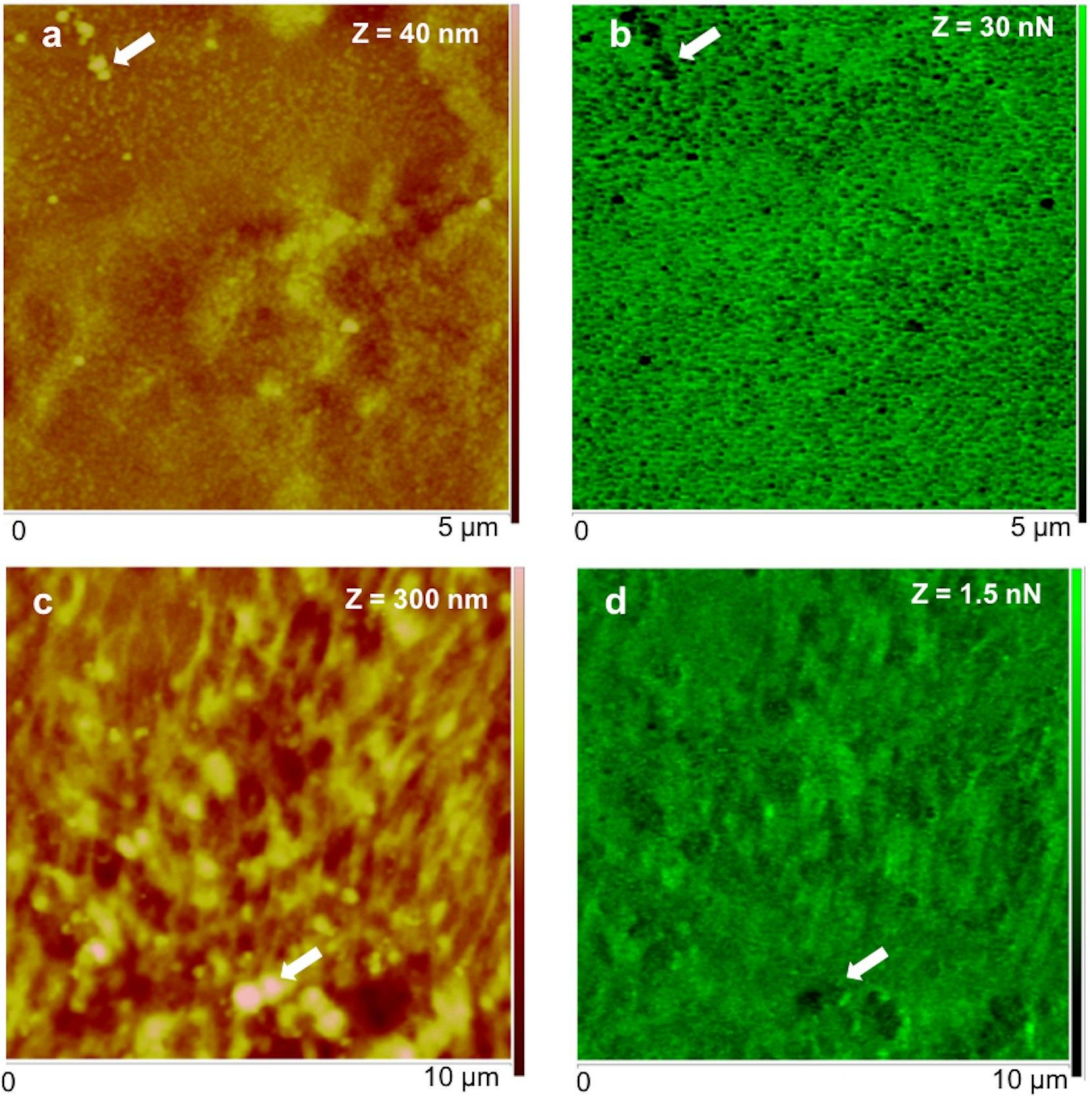
Peak force tapping AFM (PFT-AFM) with quantitative nanomechanical (QNM) software for analysis of height (a, c) and adhesion (b, d) of the adhesive material deposited by the tube feet of *Paracentrotus lividus* on mica obtained in air (a, b) and artificial seawater (c, d). a) Height and b) adhesion images of dry adhesive material observed in ambient air with a ScanAsyst-Air probe. c) Height and d) adhesion images of wet adhesive material observed in artificial seawater with a ScanAsyst-Fluid probe. Arrows indicate the location of some of the larger globular nanostructures assigned as being most likely adhesive secretory granules.

To evaluate the nanomechanical properties of sea urchin adhesive footprints we used the peak force tapping (PFT) method and quantitative nanomechanical (QNM) software, using calibrated tips, as described below in the Experimental section. This imaging method was successfully used in other temporary adhesives [5–6] and allows one force–distance curve to be obtained for each pixel of the topographical image that is then used by the QNM software to calculate an adhesion map. The obtained maps (Figure 5b,d) show that the adhesion force (i.e., the maximum forced needed to pull off the probe from the adhesive) is higher when the adhesive is analysed in air (in the range of 30 nN; Figure 5a,b) than under native conditions (in the range of 1.5 nN; Figure 5c,d). However, in both conditions, the bigger globular nanostructures, assigned to adhesive secretory granules, are much less adhesive (darker areas in the adhesion map) than the surrounding meshwork of interconnected threads made of minute globular nanostructures (greener areas in the adhesion map). Our results are in line with the adhesive forces reported for other temporary adhesives that range between 63 pN to 66.4 nN [1–6], for which lower adhesion forces were also obtained in fluid (milli-Q or ASW) than in air conditions. Indeed, using AFM it is expected that adhesion forces are considerably lower when performed in liquid environment. In water, the capillary effect (caused by the formation of a meniscus between the tip and the sample due to air vapour) is eliminated, thus the measured adhesion forces are usually one or two orders of magnitude lower than in air [24].

**Figure 6:**
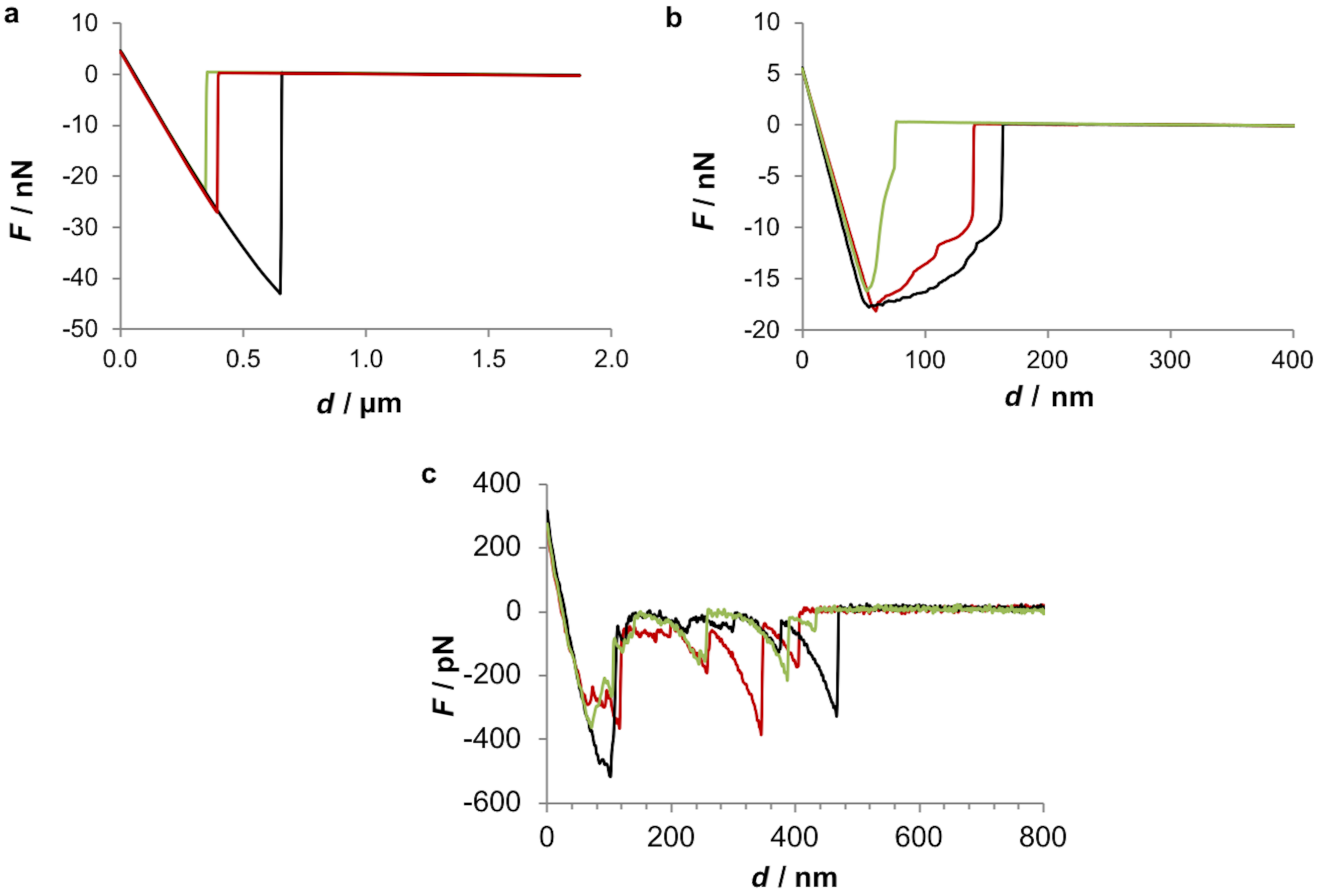
Force–distance retracting curves of the adhesive material deposited by the tube feet of *Paracentrotus lividus* on mica. The adhesive material was observed either dry (a), moist (b) or under native (c) conditions. The data was obtained either with a ScanAsyst-Air probe (a, b) or a silicon nitride probe (SNL, Bruker) (c). Different coloured lines represent three independent trials.

We also analysed individual force–distance curves obtained in the three tested conditions to get further information about the interaction between the tip and the adhesive (Figure 6). The obtained retraction curves showed that when the adhesive is probed dry, a typical force–distance curve is obtained and maximum pull-off forces can reach almost 50 nN (Figure 6a). However, when the adhesive is probed under native conditions (ASW), the resultant force curve shows a characteristic sawtooth appearance with multiple smaller pull-off forces not higher than 500 pN (Figure 6c). When a moist adhesive sample is probed (Figure 6b), an intermediate behaviour is observed, since maximum pull-off forces are around 20 nN and the shape of the pull-off curves present very small detachment events but not quite a typical saw-tooth signature.

The above-mentioned sawtooth pattern of the force–distance retracting curve was previously observed in the temporary adhesives of the marine flatworm *E. solae* and in *S. balanoides* and *B. amphitrite* barnacle cyprid larvae [1–4]. This pattern is often interpreted as the repetitive breaking of sacrificial bonds within an intermolecular β-sheet as protein is pulled from the surface of the adhesive fibrils and the “hidden length” of the amino acid chain is extended [1]. In *E. solae* the sawtooth peaks observed in the retracting force–extension curve were equally spaced (15.5 ± 4.2 nm), indicating an underlying repetitive structural unit within the adhesive. According to the authors, since the adhesive is proteinaceous in nature, this repetitive hidden length corresponds approximately to the number of amino acids involved in the intermolecular β-sheet, which in that case was 43 amino acids [1]. In the present study this periodicity between detachment events was not observed.

**Figure 7:**
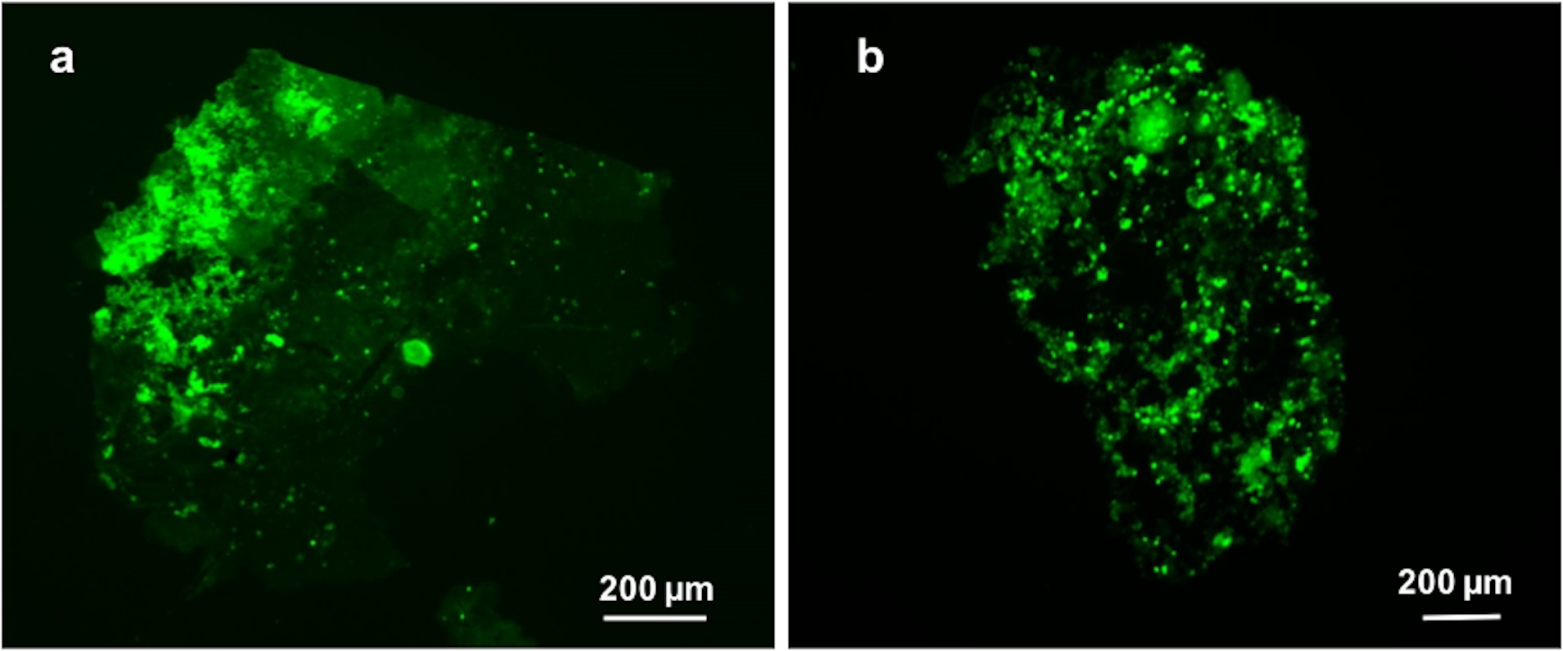
a) Histological staining of the adhesive material deposited by the tube feet of *Paracentrotus lividus* on glass with thioflavin-T and b) illustration of different adhesive footprints.

The suggested amyloid nature of sea urchin temporary adhesives was further investigated using thioflavin-T, with which a clear positive labelling of the adhesive footprints was obtained, especially in the thicker areas of the adhesive, although the labelling of the thinner adhesive areas is also visible (Figure 7a,b). Nevertheless, it should be stressed that although thioflavin-T has been widely used to investigate amyloid formation since 1989, this stain does not recognize amyloid fibrils per se, but instead binds to the molecular groove typically found on the surface of amyloid fibrils. Therefore, besides binding to amyloid proteins and peptides, thioflavin-T can also bind DNA, some polysaccharides and β-sheet-rich proteins [25].

Taken together, our results stress the importance of probing marine adhesives under native conditions to obtain more representative results and indicate that sea urchin adhesives might contain amyloid quaternary protein structures or β-sheet-rich proteins, but this should be further confirmed by complementary methods such as circular dichroism spectroscopy, TEM or Fourier transform infrared spectroscopy. As previously discussed by other authors, this mechanism of sacrificial bonds and hidden length is not necessarily associated to adhesion per se but instead influences the cohesive strength of marine adhesives [1], being thus advantageous for sea urchins because of the extra energy required to pull its adhesive apart.

## Conclusion

The adhesive footprints of the sea urchin *Paracentrotus lividus* were characterized for the first time at the nanoscale level. The adhesive topography was similar in dry and native conditions being composed of a honeycomb-like meshwork of interconnected threads of globular nanostructures. In terms of adhesion forces, higher values were achieved in dry conditions, reaching up to 50 nN. Under native conditions lower adhesive forces were obtained but the adhesive behaved like a functional amyloid as evidenced by the recorded characteristic sawtooth force–extension retracting curves and positive labelling with thioflavin-T. These results suggest that sea urchin temporary adhesives most likely contain proteins with amyloid quaternary structures or rich in β-sheets that likely contribute to the increased cohesiveness.

## Experimental

### Collection and maintenance of sea urchins

Individual *Paracentrotus lividus* (Lamarck, 1816) were collected intertidally in Ericeira, Portugal. They were kept in a marine aquarium with closed circulation (18 °C, 33% salinity) and fed algae (*Laminaria* sp.). To collect the sea urchin adhesive material, the animals were overturned and the oral tube feet were allowed to adhere firmly to a freshly cleaved mica surface and then voluntarily detach, leaving the adhesive secretion on the substrate (mica for AFM and glass for histochemistry) as a footprint. All methodologies used comply with national legislation and guidelines.

### Atomic force microscopy (AFM)

The adhesive material was observed either dry, moist or under native conditions. In the first case, footprints deposited on mica were rinsed with distilled water to prevent imaging artefacts due to salt crystallization, then allowed to dry in ambient air for at least 24 h and further dried under N_2_ flux prior to imaging. In the second case, fresh footprints deposited on mica were rinsed with distilled water, slightly dried with N_2_ flux and immediately observed. In the third case, fresh footprints deposited on mica were maintained and observed under native conditions (immersed in filtered artificial seawater).

AFM imaging was carried out in a multimode 8 HR (Bruker) device, equipped with an optical microscope (OMV) with a 10× objective and coupled to a video camera, using peak force tapping AFM (PFT-AFM) mode and the quantitative nanomechanical software (QNM; Bruker). The images were acquired at a scan rate of ≈1.0 Hz, in ambient conditions (≈21 °C), using a ScanAsyst-air probe (Bruker) with a spring constant of 0.44 N/m. Images obtained in filtered artificial seawater were carried out in a fluid cell using either a ScanAsyst-fluid probe (Bruker) with a spring constant of 1.12 N/m or a silicon nitride cantilever with a silicon tip (SNL, Bruker) with a spring constant of 0.12 N/m.

All of the above-mentioned probes were calibrated on a stiff sample, to access tip deflection sensitivity, followed by thermal tuning to determine the spring constant.

At least five sea urchin adhesive footprints samples were imaged in each condition and representative data were selected.

### Histochemical staining

Binding of the benzothiazole dye thioflavin-T (Sigma-Aldrich) to amyloid structures was detected by direct staining of the adhesive footprints collected on glass slides. Footprints were incubated with 10 μM aqueous thioflavin-T for 5 min, washed with milli-Q water, and observed immediately with an Olympus BX60 fluorescent microscope, equipped with a 10× 0.4 NA lens and a Hamamatsu Orca R2 monochrome camera. The emission characteristics of thioflavin-T dye binding to amyloid was previously determined [26] where a peak around 550 nm (after excitation at 488 nm) and emission over 505–620 nm was observed.
